# The Assessment of Trait Emotional Intelligence: Psychometric Characteristics of the TEIQue-Full Form in a Large Italian Adult Sample

**DOI:** 10.3389/fpsyg.2018.02786

**Published:** 2019-01-17

**Authors:** Antonio Chirumbolo, Laura Picconi, Mara Morelli, K. V. Petrides

**Affiliations:** ^1^Faculty of Medicine and Psychology, Department of Psychology, Sapienza University of Rome, Rome, Italy; ^2^Department of Psychological, Health, and Territorial Sciences, D’Annunzio University of Chieti–Pescara, Chieti, Italy; ^3^Department of Developmental and Social Psychology, Sapienza University of Rome, Rome, Italy; ^4^London Psychometric Laboratory, University College London, London, United Kingdom

**Keywords:** Trait Emotional Intelligence, dimensionality, reliability, incremental validity, Big Five, depression, anxiety, TEIQue

## Abstract

Trait Emotional Intelligence (or trait emotional self-efficacy) is a constellation of emotional perceptions assessed through questionnaires and rating scales (Petrides et al., 2007b). This paper examined the psychometric features of the Trait Emotional Questionnaire Full Form (TEIQue-FF; Petrides, 2009b) in the Italian context. Incremental validity in the prediction of depression and anxiety was also tested with respect to the Big Five. Participants were 1343 individuals balanced for gender (690 females and 653 males) whose mean age was 29.65 years (*SD* = 13.64, range 17–74 years). They completed a questionnaire battery containing the TEIQue and measures of the Big Five, depression, and anxiety (both trait and state). Results indicated that the performance of the TEIQue-FF in the Italian context was comparable to the original United Kingdom version as regards its reliability and factor structure. Moreover, the instrument showed incremental validity in the prediction of depression and state-trait anxiety after controlling for the Big Five.

## Introduction

In the last decades, a relevant body of research has focused on the concept of emotional intelligence which was applied in several domains such as educational, organizational, and clinical psychology (Petrides et al., 2016). In the literature, there is a clean-cut conceptual distinction between *ability* emotional intelligence and *trait* emotional intelligence. In the first case, emotional intelligence was conceived as a cognitive-emotional ability assessed via performance-based tests, whereas in the second case emotional intelligence was conceived as a personality trait often referred to as emotional self-efficacy and assessed via self-report instruments (Petrides and Furnham, 2000).

Trait Emotional Intelligence (EI) is defined as a constellation of emotional perceptions assessed through questionnaires and rating scales (Petrides et al., 2007b). Based on a content analysis of early models of EI and cognate constructs, such as alexithymia, affective communication, emotional expression, and empathy, Petrides (2001) identified 15 distinct aspects that would frame the dimensional domain of the trait EI construct as assessed by the Trait Emotional Intelligence Questionnaire (TEIQue). More specifically, the TEIQue taps 13 different facets grouped into four second-order factors named *well-being*, *self-control*, *emotionality*, and *sociability*. Two additional facets (*adaptability* and *self-motivation*) contribute directly to the global trait EI score. In Table 1, a brief definition and a sample item for each of the facets were provided (for a detailed TEIQue description see Petrides, 2009a).

**Table 1:** The sampling domain of trait emotional intelligence in adults.

Facets	Definition
Adaptability	Being flexible and willing to adapt to new conditions
Assertiveness	Being frank, straightforward, and prepared to stand up for one’s own rights
Emotion perception (self and others)	Being clear about their own and other people’s feelings
Emotion expression	Being capable of communicating their feelings to others
Emotion management (others)	Being capable of affecting other people’s feelings
Emotion regulation	Being capable of controlling one’s own emotions
Impulse control	Being reflective and less likely to surrender to one’s own drives
Relationships	Being capable of having satisfying personal relationships
Self-esteem	Being successful and self-confident
Self-motivation	Being driven and unlikely to surrender in front of difficulty
Social awareness	Being talented in networking with good social skills
Stress management	Being capable of cope with pressure and regulate stress
Trait empathy	Being capable of taking someone else’s perspective
Trait happiness	Being cheerful and satisfied with one’s own life
Trait optimism	Being confident and positive


As regards the location of trait EI in the factorial space of personality and its discriminant validity, factor analyses have demonstrated that trait EI facets define a distinct oblique factor in Giant Three and Big Five factor space (Petrides et al., 2007b). This pattern of results has been replicated in different samples and cultural contexts (e.g., Petrides et al., 2010; Van der Linden et al., 2012; Pérez-González and Sanchez-Ruiz, 2014) and extended also to the HEXACO model of personality (Veselka et al., 2009a). In addition, another investigation pointed out that correlations between trait EI and the Big Five are sizable, stable, and partly influenced genetically (Vernon et al., 2008). More recent studies reported consistent overlap between the so-called “General Factor of Personality” (GFP) and trait EI (e.g., Veselka et al., 2009a,b; Pérez-González and Sanchez-Ruiz, 2014). In general, findings showed that the highest loadings in the GFP came from trait EI and that the correlation between trait EI and the GFP is very strong and genetically influenced, remaining high even after controlling for social desirability (Van der Linden et al., 2012, 2017, 2018).

There is also a growing body of evidence, including from meta-analyses (Andrei et al., 2016a), showing the criterion and incremental validity of TEIQue in predicting a wide range of criteria over and above the Big Five and the Giant Three, such as life satisfaction, emotional reactivity, coping styles, depression, loneliness, rumination, and personality disorders (e.g., Austin et al., 2005; Mikolajczak et al., 2007a; Petrides et al., 2007a; Szczygieł and Mikolajczak, 2017). When compared to other instruments measuring EI, the TEIQue has been found to be the best predictor of multiple psychological criteria, at the same time showing incremental validity beyond age, gender, the Big Five, and the other two EI measures (Gardner and Qualter, 2010). A meta-analysis revealed that trait EI is more strongly associated than ability EI with health (Martins et al., 2010) and that the TEIQue is the best predictor of health outcomes than all other variables.

### The Present Study

The TEIQue has been translated in many languages and proved to be reliable and valid in different cultural and linguistic contexts (e.g., Mikolajczak et al., 2007a; Freudenthaler et al., 2008; Martskvishvili et al., 2013; Gökçen et al., 2014; Stamatopoulou et al., 2018). The present study aimed to test the psychometric properties of the TEIQue Full Form, investigating its reliability, factor structure, and construct validity using an Italian-speaking sample. Previous assessments of the TEIQue in Italy have principally focused on the short form of the instrument and have, mainly or exclusively, relied on adolescents or students, so that its generalizability remains limited (e.g., Andrei et al., 2014; Andrei et al., 2016b; Di Fabio et al., 2016). Accordingly, our research sought to scrutinize the psychometric features of the full form of the instrument employing a large sample of adults.

More specifically, we firstly aimed to assess the underlying TEIQue factor structure. The TEIQue-FF was not designed to be factor analyzed at the item level. As Petrides (2009b, p. 89) pointed out: “The TEIQue is based on a combination of the construct-oriented and inductive approaches to scale construction (Hough and Paullin, 1994). The instrument was designed to be factor analyzed at the facet level in order to avoid the problems associated with item-level factor analysis (Bernstein and Teng, 1989). Its higher-order structure is explicitly hypothesized as oblique, in line with conceptions of multifaceted constructs. Consequently, factor overlap as well as cross-loadings are to be expected and provide the justification for aggregating factor scores into global trait EI.” Therefore, in the present study, the factor analyses were carried out at the facet level as in other papers in the literature.

Specifically, the TEIQue factor structure was examined applying an Explorative Structural Equation Modeling approach (ESEM; Asparouhov and Muthén, 2009). ESEM represents a data analytic strategy suitable for investigating the latent structure underlying multi-dimensional personality tests and has been fruitfully applied also to assess the factor structure of the TEIQue Short Form (Perera, 2015). In contrast to the common confirmatory approach, in the ESEM frame work both primary and non-target loadings are freely estimated and factors can be rotated (Asparouhov and Muthén, 2009; Morin et al., 2013; Marsh et al., 2014). In this regard, ESEM provides a less restrictive test for examining the latent factor structure that can satisfactorily account for the complexity of multidimensional instruments. ESEM is an integration of Explorative Factor Analysis (EFA) within the general structural equation modeling framework (Asparouhov and Muthén, 2009). Compared to EFA, ESEM technique has the compelling advantage of the statistical features of SEM, like, for example, the possibility of estimating model fit indexes (Morin et al., 2013; Marsh et al., 2014).

The reliability of the 20 TEIQue variables (15 facets, 4 factors, and the global score) and gender differences in trait scores were also assessed. Subsequently, we investigated the incremental validity over the Big Five of the Italian adaptation of the TEIQue in relation to the prediction of depression and anxiety. It was expected that trait EI would remain a reliable negative predictor of depression and anxiety in the presence of the Big Five traits.

## Materials and Methods

### Participants

Participants were 1343 Italian adults balanced for gender (690 females and 653 males). Average age was 29.65 years (*SD* = 13.64, range = 17–74 years). As regards educational level, about 7% of participants completed junior high-school, 85% completed high-school, and 8% had a university degree. Concerning occupation, 7.1% of participants were blue-collar workers, 16.4% white-collar workers, 7.1% self-employed individuals, 4.7% housewives, and 53.5% university students (about 11.3% reported other occupations).

Data were collected by first-year psychology students in introductory statistics courses at the D’Annunzio University of Chieti–Pescara. Each student was requested to collect questionnaires from two to four people, equally balanced for age and gender^1^. Students were blind regarding research hypotheses and were instructed on how to administer the questionnaires. After data collection, students were briefed on the general aim of the research. Participants received written instructions about how to fill the questionnaire on the first page of the booklet. Instructions guaranteed for anonymity of responses and pointed out that there were no “correct” or “wrong” answer for questionnaire items. For a similar procedure, see Caprara et al. (2006).

The study was approved by the Psychological Science Departmental ethics committee at the D’Annunzio University of Chieti–Pescara.

### Measures

Trait EI was operationalized through the Italian adaptation of the TEIQue-FF (Petrides, 2009a,b). The TEIQue-FF comprises 153 brief statements rated by participants on a 7-point scale, ranging from completely disagree (1) to completely agree (7). The TEIQue-FF consists of 13 facets (Table 1) clustered under four-factors: well-being, self-control, emotionality, and sociability. Two additional facets (namely adaptability and self-motivation) contribute directly to the global trait EI score but are not part of the factors (for a detailed description of the TEIQue, see Petrides, 2009a).

Personality traits were assessed via the Big Five Questionnaire (BFQ-2; Caprara et al., 1993, 2007) which comprises 134 items rated on a 5-point Likert scale (1 = very false for me, 5 = very true for me). The BFQ was been shown to be a valid and reliable measure of the Big Five traits in large samples of Italian respondents as well as in cross-cultural comparisons (e.g., Caprara et al., 2000). In the present study, the internal consistencies of the five traits were 0.84 for Extraversion, 0.88 for Agreeableness, 0.86 for Conscientiousness, 0.87 for Openness, and 0.92 for Emotional Stability.

The Italian version of Beck Depression Inventory-II (BDI-II; Beck et al., 2006) was used to assess depression. The BDI is a 21-item self-report inventory designed to assess the presence and severity of depressive symptoms. Each item is rated on a 4-point Likert-type scale, ranging from 0 to 3, based on the severity of depressive symptoms experienced over the last 2 weeks. Each item presents a list of four statements arranged in increasing severity about a particular symptom of depression. The total score ranges from 0 to 63, with higher scores indicating more severe depressive symptoms. The psychometric features of the scale are supported in clinical and non-clinical samples by an extensive literature (e.g., Arbisi, 2001). In this study, the Cronbach’s alpha of the BDI-II was 0.87.

The Italian version of the State-Trait Anxiety Inventory (STAI-Y) was employed to measure trait and state anxiety (Spielberger, 1989). This measure is commonly used in clinical settings to diagnose anxiety as distinct from depression. The Y form consists of 20 items targeting Trait anxiety and 20 targeting State anxiety. State anxiety items include: “I am tense; I am worried” and “I feel calm; I feel secure.” Trait anxiety items include: “I worry too much over something that really doesn’t matter” and “I am content; I am a steady person.” All items are rated on a 4-point Likert scale (e.g., from “Almost Never” to “Almost Always”). The total score ranges from 20 to 80, with higher scores indicating greater anxiety. In the present study, the STAI-State (α = 0.94) and the STAI-Trait (α = 0.90) demonstrated excellent internal consistency.

All 1343 participants completed the TEIQue-FF. For the validity investigations, a subsample of 409 participants also completed the measures of the Big Five, depression, and trait and state anxiety. The mean age for the subsample was 28.06 years (*SD* = 13.07, range = 17–66 years), while the gender distribution was 221 females and 188 males.

### Data Analysis

The TEIQue factor structure was examined via ESEM (Asparouhov and Muthén, 2009). Besides the chi-square, the fit of the factorial model was evaluated via the Comparative Fit Index (CFI), the Standardized Root Mean Square Residual (SRMR), and the root mean square error of approximation (RMSEA). The following guidelines, derived from existing literature (Browne and Cudeck, 1993; Hu and Bentler, 1999; Marsh et al., 2004), were used to assess the adequacy of model fit: *CFI* > 0.90, *RMSEA* < 0.08, and *SRMR* < 0.10 were considered acceptable fit, while *CFI* > 0.95, *RMSEA* < 0.05, and *SRMR* < 0.08 were deemed to reflect an excellent fit.

Internal consistencies for all TEIQue variables were estimated using Cronbach’s alpha. Descriptive statistics and general distributional properties of the scales were also assessed. A one-way ANOVA was carried out to investigate gender differences in trait EI scores. Finally, the incremental validity of trait EI in the prediction of depression and anxiety beyond the Big Five personality traits was tested through hierarchical regression analyses conducted at the factor level of the TEIQue.

Statistical data analyses were conducted with MPlus8 and SPSS 24.

## Results

### Factor Structure of the TEIQue-FF

The aforementioned ESEM analysis was applied to the 13 TEIQue-FF Facets^2^. Standardized factor loading estimates from the retained ESEM four correlated traits model, with oblique rotation, are shown in Table 2. Despite one cross-loading between Self-esteem and Sociability, the four factors were substantively identical to the original United Kingdom structure (Petrides, 2009a) and were thus labeled accordingly: Well-Being, Self-Control, Emotionality, and Sociability. The fit of the retained model was excellent, χ^2^(32) = 331.42, *p* < 0.001, *CFI* = 0.95, *RMSEA* = 0.08, *SRMR* = 0.03, and in line with those reported by other authors who have conducted similar analyses (Perera, 2015). In the final solution, the four factors were positively and significantly correlated (Table 3). Factor inter-correlations were generally above 0.40, with the exception of Self-Control that had somewhat lower correlations with Sociability (0.20) and Emotionality (0.22).

**Table 2:** Factor loadings for the retained ESEM four correlated traits model.

	Well-Being	Self-Control	Emotionality	Sociability
Trait happiness	**0.81**	-0.07	0.09	-0.00
Trait optimism	**0.77**	0.09	-0.03	0.08
Self-esteem	**0.37**	0.07	-0.00	0.39
Emotion regulation	0.00	**0.69**	-0.04	0.06
Impulse control	-0.02	**0.34**	0.23	0.04
Stress management	0.19	**0.62**	0.07	-0.03
Trait empathy	-0.12	0.03	**0.54**	-0.01
Emotion perception	0.03	0.01	**0.54**	0.09
Emotion expression	0.25	-0.20	**0.54**	0.16
Relationships	0.23	0.02	**0.34**	-0.06
Emotion management	-0.12	-0.01	0.14	**0.58**
Assertiveness	0.02	-0.02	-0.05	**0.66**
Social awareness	0.06	0.02	0.17	**0.57**


*Coefficients that should theoretically define each factor are in boldface.*

**Table 3:** TEIQue-FF factor intercorrelations.

Factor	Well-Being	Self-Control	Emotionality	Sociability
Well-being	–			
Self-control	0.32^∗∗^	–		
Emotionality	0.41^∗^	0.22^∗^	–	
Sociability	0.43^∗∗^	0.20^∗∗^	0.47^∗∗^	–


*^∗^*p* < 0.01, ^∗∗^*p* < 0.001.*

### Descriptive Statistics, Reliability, and Gender Differences

Descriptive statistics, number of items, and internal consistencies for the TEIQue-FF facets, factors and global score, are given in Table 4 for the total sample and for men and women separately in Table 5.

**Table 4:** Descriptives for the TEIQue-FF facet, factor, and global scores (total sample *N* = 1343).

	Items	α	R_it(mean)_	Skewness	Kurtoses	Mean	*SD*
**Facets**							
Self-esteem	11	0.80	0.47	-0.36	-0.06	4.98	0.86
Emotion expression	10	0.85	0.56	-0.14	-0.42	4.39	1.17
Self-motivation	10	0.69	0.36	-0.11	-0.34	4.95	0.79
Emotion regulation	12	0.76	0.40	0.09	0.05	4.10	0.85
Trait happiness	8	0.84	0.59	-0.59	-0.27	5.59	0.98
Trait empathy	9	0.68	0.37	-0.05	-0.01	4.83	0.81
Social awareness	11	0.77	0.43	-0.11	-0.33	4.78	0.86
Impulse control	9	0.70	0.38	-0.14	-0.36	4.66	0.95
Emotion perception	10	0.68	0.34	-0.07	-0.21	4.90	0.80
Stress management	10	0.74	0.41	-0.17	0.04	4.34	0.92
Emotion management	9	0.68	0.36	-0.07	-0.14	4.62	0.87
Trait optimism	8	0.78	0.49	-0.11	-0.56	4.97	0.99
Relationships	9	0.55	0.26	-0.27	-0.28	5.41	0.73
Adaptability	9	0.63	0.32	-0.10	0.31	4.17	0.79
Assertiveness	9	0.65	0.33	-0.07	-0.20	4.71	0.86
**Factors**							
Emotionality	38	0.70	0.50	0.10	-0.35	4.88	0.65
Self-control	31	0.69	0.51	0.11	-0.08	4.37	0.71
Sociability	29	0.78	0.62	0.06	-0.24	4.70	0.72
Well-being	27	0.82	0.68	-0.32	-0.48	5.18	0.81
Global trait EI		0.86	0.51	0.15	-0.33	4.76	0.52


*R_*it*(*mean*)_, mean inter-item correlation.*

**Table 5:** Descriptives and gender difference tests for the TEIQue-FF.

Females (*N* = 690)						Males (*N* = 653)					
			
	Mean	*SD*	α	R_it(mean)_	Skewness	Kurtoses	Mean	*SD*	α	R_it(mean)_	Skewness	Kurtoses	*F*
Self-esteem	4.84	0.91	0.82	0.50	-0.29	-0.20	5.13	0.77	0.75	0.42	-0.29	-0.13	39.59^∗∗∗^
Emotion expression	4.43	1.22	0.87	0.59	-0.25	-0.49	4.34	1.11	0.83	0.53	-0.01	-0.31	2.38
Self-motivation	4.92	0.80	0.69	0.36	-0.17	-0.16	4.98	0.79	0.69	0.37	-0.04	-0.57	2.11
Emotion regulation	3.87	0.79	0.72	0.36	0.02	-0.15	4.35	0.84	0.76	0.40	0.07	0.18	113.33^∗∗∗^
Trait happiness	5.56	1.01	0.86	0.62	-0.53	-0.49	5.61	0.95	0.82	0.56	-0.66	0.01	0.74
Trait empathy	5.00	0.77	0.66	0.35	0.01	-0.34	4.65	0.82	0.68	0.36	-0.03	0.23	64.74^∗∗∗^
Social awareness	4.72	0.86	0.77	0.42	-0.03	-0.28	4.84	0.87	0.78	0.44	-0.20	-0.33	7.11^∗∗^
Impulse control	4.63	0.92	0.68	0.35	-0.06	-0.42	4.69	0.98	0.73	0.40	-0.23	-0.30	1.11
Emotion perception	4.96	0.80	0.68	0.34	0.02	-0.36	4.83	0.80	0.69	0.35	-0.17	-0.11	8.90^∗∗^
Stress management	4.12	0.90	0.72	0.39	-0.20	0.01	4.58	0.89	0.74	0.40	-0.14	0.04	86.97^∗∗∗^
Emotion management	4.60	0.87	0.68	0.36	-0.05	-0.29	4.64	0.87	0.68	0.36	-0.09	0.04	0.90
Trait optimism	4.85	1.01	0.79	0.49	0.00	-0.57	5.11	0.95	0.77	0.47	-0.21	-0.49	23.00^∗∗∗^
Relationships	5.46	0.71	0.52	0.25	-0.38	-0.22	5.35	0.74	0.57	0.28	-0.16	-0.30	8.22^∗∗^
Adaptability	4.10	0.76	0.61	0.30	-0.11	0.35	4.24	0.81	0.65	0.34	-0.11	0.27	9.66^∗∗^
Assertiveness	4.63	0.87	0.66	0.33	-0.12	-0.36	4.80	0.85	0.64	0.32	0.01	-0.07	12.26^∗∗∗^
**Factors**													
Emotionality	4.97	0.64	0.67	0.47	0.15	-0.45	4.79	0.65	0.73	0.53	0.08	-0.30	24.20^∗∗∗^
Self-control	4.21	0.67	0.65	0.47	0.09	0.00	4.54	0.72	0.71	0.53	0.06	-0.17	75.04^∗∗∗^
Sociability	4.65	0.72	0.78	0.61	0.13	-0.37	4.76	0.72	0.78	0.62	-0.01	-0.05	8.08^∗∗^
Well-being	5.08	0.84	0.82	0.68	-0.23	-0.55	5.28	0.77	0.83	0.69	-0.38	-0.40	20.23^∗∗∗^
Global trait EI	4.71	0.51	0.85	0.49	0.16	-0.32	4.80	0.53	0.88	0.53	0.12	-0.35	11.20^∗∗^


*^∗∗^*p* < 0.01, ^∗∗∗^*p* < 0.001; R_it(mean)_, mean inter-item correlation.*

All TEIQue variables (facets, factors, and global score) had reasonably normal distributions. None of the variables had a skew or kurtosis greater than 1 (in absolute value). Eight of the 15 facets had high alphas (between 0.70 and 0.85; Table 4), six showed moderate levels (between 0.63 and 0.68), and one (Relationships) showed a low level (0.55). Reliabilities were satisfactory for all four TEIQue factors: Well-being (0.82), Self-control (0.69), Sociability (0.78), and Emotionality (0.70). Corrected item-total correlations ranged from 0.26 (Relationships) to 0.59 (Trait happiness) and from 0.50 (Emotionality) to 0.68 (Well-being). The reliability of the global trait EI score was high (α = 0.86).

With respect to gender differences, significant differences were observed at the facet, factor, and global levels of the instrument (see Table 5). Means and standard deviations as well as gender-specific alphas, for the 15 facets, 4 factors, and global trait EI can be seen in Table 4. Males scored significantly higher on the facets of Self-Esteem, Emotion Regulation, Social Awareness, Stress Management, Trait optimism, Adaptability, and Assertiveness, as well as on the factors of Self-Control, Sociability, Well-being, and global trait. Conversely, females scored significantly higher on the facets of Trait empathy, Emotion perception, and Relationships, as well as on the factor of Emotionality.

### Incremental Validity

In order to test the validity of the TEIQue, a subsample of 409 participants also completed measures of the Big Five, depression and anxiety as reported in the method section. Correlations among these variables are presented in Table 6.

**Table 6:** Intercorrelations for key variables in the study.

	1	2	3	4	5	6	7	8	9	10	11
1. Trait EI wellbeing	–										
2. Trait EI self-control	0.42^∗∗^	–									
3. Trait EI emotionality	0.43^∗∗^	0.27^∗∗^	–								
4. Trait EI sociability	0.52^∗∗^	0.26^∗∗^	0.46^∗∗^	–							
5. Energy	0.43^∗∗^	0.09^∗^	0.20^∗∗^	0.58^∗∗^	–						
6. Openness	0.18^∗∗^	0.17^∗∗^	0.32^∗∗^	0.28^∗∗^	0.33^∗∗^	–					
7. Agreeableness	0.21^∗∗^	0.15^∗∗^	0.52^∗∗^	0.21^∗∗^	0.15^∗∗^	0.45^∗∗^	–				
8. Conscientiousness	0.26^∗∗^	0.26^∗∗^	0.25^∗∗^	0.24^∗∗^	0.38^∗∗^	0.34^∗∗^	0.38^∗∗^	–			
9. Emotional stability	0.42^∗∗^	0.68^∗∗^	0.14^∗∗^	0.19^∗∗^	0.08	0.11^∗∗^	0.04	0.05	–		
10. Depression	-0.59^∗∗^	-0.43^∗∗^	-0.23^∗∗^	-0.27^∗∗^	-0.24^∗∗^	-0.12^∗∗^	-0.06	-0.15^∗∗^	-0.50^∗∗^	–	
11. State anxiety	-0.55^∗∗^	-0.48^∗∗^	-0.26^∗∗^	-0.34^∗∗^	-0.23^∗∗^	-0.13^∗∗^	-0.13^∗∗^	-0.16^∗∗^	-0.55^∗∗^	0.64^∗∗^	–
12. Trait anxiety	-0.71^∗∗^	-0.48^∗∗^	-0.28^∗∗^	-0.40^∗∗^	-0.36^∗∗^	-0.16^∗∗^	-0.07	-0.13^∗∗^	-0.60^∗∗^	0.68^∗∗^	0.75^∗∗^


*^∗^*p* < 0.05, ^∗∗^*p* < 0.01.*

As expected, the four TEIQue factors were all significantly and negatively correlated with depression and both types of anxiety, confirming that TEIQue scores are strongly related to mental health variables (Martins et al., 2010; Rudenstine and Espinosa, 2018).

To examine the incremental validity of the TEIQue-FF factors beyond the Big Five, we conducted three separate hierarchical regression analyses^3^. For each criterion, the Big Five traits were entered as a first block of predictors, followed by the four TEIQue factors as a second block. This data analytic strategy aimed at examining the contributions of the Big Five traits in predicting the criteria (depression and anxiety) at step 1, and the incremental variance accounted by the TEIQue-FF traits at step 2 (*R*^2^ change). If the TEIQue-FF traits have incremental validity over the Big Five ones, then they would be expected to explain a significant amount of additional variance at step 2 (that is, the *R*^2^ change would be statistically significant).

Concerning depression, the Big Five traits collectively explained 30% of the variance, *R*^2^ = 0.30, *p* < 0.001. When the TEIQue traits were included in the second step of the hierarchical regression, an additional 16% of variance was accounted for, *R*^2^ = 0.46, *R*^2^_change_ = 0.16, *p* < 0.001. Regression coefficients for the second step are reported in Table 7.

**Table 7:** Step 2 results for hierarchical regression of depression on the four TEIQue factors and the Big Five.

Depression					
	*b*	*SE*	*Beta*	*t*	*p*
	
Trait EI well-being	-4.307	0.450	-0.480	-9.578	0.000
Trait EI self-control	-0.234	0.606	-0.022	-0.387	0.699
Trait EI emotionality	-0.646	0.597	-0.055	-1.082	0.280
Trait sociability	0.364	0.586	0.035	0.620	0.535
Extraversion	1.708	0.856	0.107	1.996	0.047
Agreeableness	2.350	0.781	0.143	3.007	0.003
Conscientiousness	-0.981	0.717	-0.062	-1.369	0.172
Emotional stability	-3.726	0.674	-0.301	-5.527	0.000
Open mindedness	-1.115	0.614	-0.079	-1.816	0.070


Concerning state anxiety, the Big Five traits collectively explained 32% of the variance, *R*^2^ = 0.32, *p* < 0.001. The TEIQue traits subsequently explained a further 9% of criterion variance, *R*^2^ = 0.41, *R*^2^_change_ = 0.09, *p* < 0.01. Regression coefficients for the second step are reported in Table 8.

**Table 8:** Step 2 results for hierarchical regression of state anxiety on the four TEIQue factors and the Big Five.

State anxiety					
	*b*	*SE*	*Beta*	*t*	*p*
	
Well-being	-4.206	0.691	-0.316	-6.086	0.000
Self-control	-1.355	0.931	-0.086	-1.455	0.146
Emotionality	-0.424	0.917	-0.024	-0.462	0.644
Sociability	-1.130	0.900	-0.073	-1.255	0.210
Extraversion	1.413	1.314	0.060	1.075	0.283
Agreeableness	-0.085	1.201	-0.003	-0.071	0.944
Conscientiousness	-1.048	1.101	-0.045	-0.952	0.342
Emotional stability	-6.054	1.035	-0.330	-5.848	0.000
Open mindedness	-0.025	0.943	-0.001	-0.026	0.979


Concerning trait anxiety, the Big Five traits collectively explained 45% of the variance, *R*^2^ = 0.45, *p* < 0.001. The TEIQue traits subsequently explained an additional 21% of criterion variance, *R*^2^ = 0.66, *R*^2^_change_ = 0.21, *p* < 0.001. Regression coefficients for the second step are reported in Table 9.

**Table 9:** Step 2 results for hierarchical regression of trait anxiety on the four TEIQue factors and the Big Five.

Trait anxiety					
	*b*	*SE*	*Beta*	*t*	*p*
	
Trait EI well-being	-6.171	0.466	-0.524	-13.242	0.000
Trait EI self-control	-1.086	0.628	-0.078	-1.728	0.085
Trait EI emotionality	-1.195	0.619	-0.078	-1.931	0.054
Trait EI sociability	0.420	0.608	0.031	0.690	0.490
Extraversion	-1.598	0.887	-0.076	-1.802	0.072
Agreeableness	2.240	0.810	0.104	2.766	0.006
Conscientiousness	-0.520	0.743	-0.025	-0.700	0.484
Emotional stability	-5.331	0.699	-0.328	-7.628	0.000
Open mindedness	-0.186	0.637	-0.010	-0.292	0.770


As can be noted, the TEIQue-FF showed clear predictive capability in the presence of the Big Five, incrementally explaining significant proportions of variance across all three criteria.

## Discussion

The present study scrutinized the psychometric characteristics of the Italian TEIQue-FF by investigating the instrument’s distributional properties, reliability, factor structure, and gender differences. Moreover, criterion and incremental validity were established by demonstrating the ability of the four TEIQue factors to predict mental health (i.e., absence of depression and anxiety) over and above the Big Five. The distributional properties of the Italian version of the TEIQue-FF were satisfactory, with all of its variables showing near-normal distributions with means and standard deviations comparable to those obtained for the original scale (Petrides, 2009a).

In general, the reliability of TEIQue scales reached satisfactory levels although, for the Italian version, they appear to be a little lower than for other translations (e.g., Mikolajczak et al., 2007a; Freudenthaler et al., 2008; Petrides, 2009a). However, the only facet with a low internal consistency was Relationships, which has displayed lower alpha values in many countries, such as the United Kingdom (Petrides, 2009a), France (Mikolajczak et al., 2007a), Georgia (Martskvishvili et al., 2013), Germany (Freudenthaler et al., 2008), and Serbia (Joliæ-Marjanoviæ and Altaras-Dimitrijeviæ, 2014) as well as in the autonomous community of Catalonia (Aluja et al., 2016). More important, the four-factor structure of the TEIQue emerged clearly from the Italian data, replicating robust, and consistent results from many different countries around the world. In regards to the validity of the instrument, the TEIQue factors were significantly and negatively associated with depression and the two types of anxiety (state and trait). In line with previous research, these results persisted even after controlling for the Big Five personality dimensions, thus confirming the excellent incremental validity of the TEIQue (e.g., Siegling et al., 2015; Andrei et al., 2016a).

Despite the robust psychometric properties of the Italian version of the TEIQue highlighted by this study, some limitations should be listed. Due to its length, the full form of the TEIQue may be not suitable for rapid clinical screenings or for research designs where there is limited availability of time and space for data collection and wherein trait EI is not a central variable. In those cases, the short form could be more appropriate. Conversely, however, the full form of the TEIQue would be more useful for in-depth analyses at the factor and facet levels of the construct. The richness of information derived from the administration of the full form is most valuable, for instance, in clinical examinations, coaching assessments, or career and vocational counseling contexts. In the present study all the variables were operationalized through self-report questionnaires, which may have created common method variance that could potentially inflate construct relationships. Future research could profitably employ a multi-method approach in order to demonstrate the incremental validity of the TEIQue in relation to external criteria measured using, for example, observational methods. Moreover, the present findings are based on correlational and cross-sectional data. Thus, they cannot be used to infer causal relationships between trait EI and the criteria examined.

The TEIQue and its underlying theory of trait emotional intelligence, have a wide range of important applications in educational, clinical, and organizational contexts (see Petrides et al., 2016 for a summary of recent developments). Regarding educational implications, the TEIQue can be used to measure trait EI within school and university contexts in order to identify students who are in need of intervention programs to eradicate disruptive behavior and enhance well-being (Ruttledge and Petrides, 2012). High trait EI also appears to best advantages in relation to academic performance particularly for vulnerable groups of children, such as those with learning difficulties (Petrides et al., 2004; Mavroveli and Sánchez-Ruiz, 2011; Perera and DiGiacomo, 2013). Nevertheless, teachers and educators should also take into consideration possible maladaptive effects of trait EI. For example, students who are confident in managing and understanding others’ emotions, could attempt to exploit or manipulate their peers as happens in bullying dynamics (Sutton and Keogh, 2000).

In vocational contexts, trait EI, in general, and the TEIQue, in particular, have been linked, phenotypically as well as genetically, to vocational interests (Schermer et al., 2015). In addition, there are significant differences in the trait EI profiles of students studying different subjects in university (Sánchez-Ruiz et al., 2010). The construct has also been strongly linked to career adaptability (Merino-Tejedor et al., 2018) and career-related decision-making (Di Fabio and Saklofske, 2014; Farnia et al., 2018). It, therefore, seems clear that trait EI should be taken into consideration by career-counselors as well by people entering the workforce or those considering a career change.

Trait EI is also a solid predictor of important outcomes in business and organizational contexts (e.g., Mikolajczak et al., 2007b; O’Boyle et al., 2011; Miao et al., 2016; Petrides et al., 2016), which is why the TEIQue is used globally by HR practitioners and business coaches for recruitment, training, and development purposes. The adaptation and standardization of the instrument in the Italian language opens up diverse opportunities for similar applications in the Italian context.

Last, trait EI and the TEIQue, specifically, is a very strong negative predictor of psychopathology (Martins et al., 2010), a relationship that has been replicated in children (Russo et al., 2012), adolescents (e.g., Mavroveli et al., 2007), and adults (e.g., Petrides et al., 2017) alike. Therefore, the instrument, supported and interpreted through the underlying theory, can be usefully incorporated in clinical and counseling screening and intervention programs across most age groups.

In conclusion, the present study tested and demonstrated, in a large adult sample, that the TEIQue-FF shows robust psychometric properties in the Italian context, just as it has shown in many other countries around the world. It also provides further evidence of the stability of the TEIQue factors across different countries and cultures, thus offering support for the construct’s universality. This validation of the TEIQue builds on previous work in the Italian context that had been conducted on adolescents (Di Fabio, 2013), young adult samples (Di Fabio et al., 2016), or smaller samples comprising predominantly undergraduate students (Andrei et al., 2016b). Thus, the present research delivers a more solid basis for generalizability to the adult Italian population.

## Ethics Statement

This study was carried out in accordance with the recommendations of “APA ethical standards” with written informed consent from all subjects. All subjects gave written informed consent in accordance with the Declaration of Helsinki. The protocol was approved by the Psychological Science Departmental ethical committee at the D’Annunzio University of Chieti–Pescara.

## Author Contributions

AC designed the study, wrote the introduction, part of results and discussion, run the analyses and revised the manuscript. LP designed the study, collected the data, run the analyses and wrote most of the result section. MM wrote part of the discussion. KP supervised the entire project, wrote several part of the manuscript, and deeply revised all the manuscript.

## Conflict of Interest Statement

The authors declare that the research was conducted in the absence of any commercial or financial relationships that could be construed as a potential conflict of interest. The handling Editor declared a past co-authorship with one of the authors KP.

## Footnotes

1 For similar applications of the snowball data collection procedure in personality research, see Caprara et al. (2006), Hilbig et al. (2013), and Romero et al. (2015).

2 As in previous research (Aluja et al., 2016), the facets of adaptability and self-motivation were not entered into the factor analysis since they have been modeled to load directly on global trait EI (Petrides, 2009a,b).

3 These analyses were conducted at the factor level of trait EI in order to help address the inherently biased comparisons in incremental validity studies that pitch a single degree of freedom for trait EI against multiple degrees of freedom for personality (Petrides et al., 2007a).
